# Using protein language models for protein interaction hot spot prediction with limited data

**DOI:** 10.1186/s12859-024-05737-2

**Published:** 2024-03-16

**Authors:** Karen Sargsyan, Carmay Lim

**Affiliations:** Institute of Biomedical Sciences, Academia Sinica, Taipei, 115 Taiwan

**Keywords:** Protein language models, ESM-2, Protein–protein interaction, PPI-hotspot, Small datasets, Feature selection

## Abstract

**Background:**

Protein language models, inspired by the success of large language models in deciphering human language, have emerged as powerful tools for unraveling the intricate code of life inscribed within protein sequences. They have gained significant attention for their promising applications across various areas, including the sequence-based prediction of secondary and tertiary protein structure, the discovery of new functional protein sequences/folds, and the assessment of mutational impact on protein fitness. However, their utility in learning to predict protein residue properties based on scant datasets, such as protein–protein interaction (PPI)-hotspots whose mutations significantly impair PPIs, remained unclear. Here, we explore the feasibility of using protein language-learned representations as features for machine learning to predict PPI-hotspots using a dataset containing 414 experimentally confirmed PPI-hotspots and 504 PPI-nonhot spots.

**Results:**

Our findings showcase the capacity of unsupervised learning with protein language models in capturing critical functional attributes of protein residues derived from the evolutionary information encoded within amino acid sequences. We show that methods relying on protein language models can compete with methods employing sequence and structure-based features to predict PPI-hotspots from the free protein structure. We observed an optimal number of features for model precision, suggesting a balance between information and overfitting.

**Conclusions:**

This study underscores the potential of transformer-based protein language models to extract critical knowledge from sparse datasets, exemplified here by the challenging realm of predicting PPI-hotspots. These models offer a cost-effective and time-efficient alternative to traditional experimental methods for predicting certain residue properties. However, the challenge of explaining why specific features are important for determining certain residue properties remains.

**Supplementary Information:**

The online version contains supplementary material available at 10.1186/s12859-024-05737-2.

## Background

Large language models [1] are a remarkable advancement in artificial intelligence, demonstrating exceptional capabilities in understanding and generating human language. Their applications span diverse domains, including software development, education, scientific research, healthcare, finance, and law [1]. Analogous to how these models decode human language, deep-learning models known as protein language models have emerged to decipher the intricate language of life by learning the patterns embedded in protein sequences over evolutionary time [2–6]. The key differences between large language models and protein language models lie in their input data and intended applications. Whereas large language models are trained on massive textual datasets, protein language models harness vast protein sequence databases containing millions of amino acid (aa) sequences from various organisms [5]. However, certain data types such as per-residue binding free energy contributions [7] are experimentally arduous to collect, yielding limited datasets. Thus, machine learning algorithms may not extract meaningful insights from such sparse data. This study aims to explore the potential of protein language models in extracting subtle information from sparse datasets, exemplified by the limited dataset of protein–protein interaction (PPI) hotspots, defined as residues whose mutations significantly impair/abolish PPIs [7].

Protein language models, like large language models, are built upon transformer architectures for representation learning and generative modeling [8]. In the transformer architecture, the encoder component encodes aa sequences, mapping each residue in the input sequence to an N-dimensional vector. The value of N is determined by various factors, including the model size (i.e., number of parameters), the training dataset size, and available computational resources [4]. Each vector encapsulates the aa type and its surrounding sequence context. Protein language models learn patterns and relationships within input protein sequences during pre-training through self-supervised masking tasks. In this process, aa residues in the input protein sequence are randomly masked, and the training objective is to predict the identity of these masked residues based on contextual clues from the surrounding residues. The goal is to minimize an objective function, which represents the negative logarithm likelihood of the true aa residue given the masked sequence [4, 5, 9]. Currently, one of the largest protein language models is ESM-2 (Evolutionary Scale Model-2) with 15 billion parameters, trained on ~ 65 million unique protein sequences [5].

Protein language models offer several advantages: The encoder-generated vectors inherently encode a spectrum of features, encompassing biochemical aa properties, species information, structural homology at the superfamily and fold level, sequence alignment within a protein family, secondary and tertiary structures, long-range contacts, and protein design principles [4]. These learned representations can be independently utilized to train deep neural networks for various classification tasks, including the sequence-based prediction of secondary structures and long-range contacts [4], tertiary structures [5], inverse folding [10], and the impact of mutations on protein function [11–13]. For example, the representations learned ESM-2 have been used to train EMSFold [5], a model capable of predicting 3D structure using only a single sequence as input. In contrast, AlphaFold2 [14] requires time-consuming multiple sequence alignment. Thus, protein language model representations eliminated the need to search for evolutionarily related sequences to construct a multiple sequence alignment, enhancing prediction speed. This enabled proteome-level predictions and the discovery of new protein structures and functions [5, 6, 15]. Additionally, protein language models have often outperformed current prediction methods across various classification tasks [4, 5, 16]. They have shown the ability to transfer knowledge learned from sequences to improve antibody binding affinity by suggesting high-fitness aa substitutions based solely on the wild-type antibody sequence [17]. They can also design proteins by generating new protein sequences and corresponding predicted structures that fulfill user-defined constraints such as secondary structure, solvent accessibility, fold and active/binding-sites [18]. For example, the protein language model, ProGen, can generate artificial functional proteins across protein families [19].

While protein language models can capture information representing different levels of protein organization from sequence data alone, their ability to learn and extract specific residue properties from limited data remains uncertain. This is of particular interest for two key reasons. First, gathering experimental data for specific aa residue properties such as PPI-hotspots can be time-consuming and resource-intensive, resulting in limited data. Second, large language models have shown the capability to adapt effectively to new language tasks with minimal examples (so-called few-shot learners) [20]. Thus, we aim to assess the possibility of using protein language models and sparse data of a certain property to predict that property accurately. Specifically, we assessed whether protein language models can discern intricate details from a limited dataset of experimentally known PPI-hotspots that make substantial contributions to PPIs [7, 21]. Since a residue's functional role is influenced by its local environment [22], we harnessed information within encoder-generated vectors encompassing residue types and their sequence context. We propose using these vector elements as features/descriptors for machine-learning algorithms to identify PPI-hotspots solely from protein sequences. For training and validation, we employed a dataset comprising 414 experimentally determined PPI-hotspots and 504 nonhot spots. This dataset had been previously used to train an ensemble of classifiers to identify PPI-hot spots using the free protein structure [23]. The results show that a subset of randomly selected features suffices for robust PPI-hot spot prediction, with performance comparable to models using all elements of encoder-generated vectors as features. The performance of our approach is also comparable to that of a model trained on 10 residue features, which requires the free protein structure as input. Hence, protein language models can discern the few PPI-hot spots from sequence alone, underscoring their potential in deciphering protein intricacies, even when data are sparse. By following our strategy, one can train new predictors to classify protein residues into desired classes (exemplified herein with hot spots or nonhot spots) using only sequence data, thereby avoiding the need for dedicated feature engineering, even with a limited training dataset.

## Methods

### Dataset

For training and validation, we employed a dataset consisting of 414 experimentally confirmed PPI-hot spots and 504 PPI-nonhot spots. The 414 PPI-hot spots are found in 158 nonredundant proteins with free structures and were obtained from the updated PPI-Hotspot + PDB^BM(1.1)^ [7, 23]. These PPI-hot spots were derived from two sources: (i) mutations in the ASEdb [24] and SKEMPI 2.0 [25] database that resulted in a reduction of protein binding free energy by ≥ 2 kcal/mol, and (ii) mutations in UniProtKB [26] that were manually curated to significantly impair/disrupt PPIs. The 504 experimentally confirmed PPI-nonhot spots are found in 75 nonredundant proteins with free structures. They were chosen based on (i) mutations in the ASEdb [24] and SKEMPI 2.0 [25] database that did *not* alter the protein binding free energy by ≥ 0.5 kcal/mol, and (ii) mutations in UniProtKB [26] curated *not* to perturb PPIs.

### Representations learned by the ESM-2 protein language model

To capture the local environment surrounding a target residue, we considered a 101-aa sequence, spanning 50 residues on each side of the target residue. If the target residue was located near the N- or C-terminus, it was positioned at the N- or C-terminus within the 101-aa sequence. This process generated sequences with an average of 34 ± 7% similarity. The sequence length was chosen to strike a balance—it is not overly long to burden memory during computations, yet not too short to overlook information from the local protein environment. These 101-aa sequences were then fed into the ESM-2 protein language model. Among the various pretrained ESM-2 models, we chose the esm2_t33_650M_UR50D trained model as a representative due to its transformer architecture using 33 layers and 650 million parameters [5], and extensive pre-training on UniRef50 [7]. We direct the reader to reference [5] for details about the model’s training, validation, and performance metrics. The final layer of this model provided an N-dimensional (N = 1280) embedding vector for the target residue, yielding 1280 features as input for machine-learning algorithms to identify if the target residue is a PPI-hot spot or not.

### Model training and validation with full and subset sequence features

We conducted model training and validation using *all* features derived from the learned representations and another using only a subset of these features. For model training and validation, we use an automatic machine-learning (AutoML) framework, viz., AutoGluon (https://auto.gluon.ai/). We chose AutoGluon due to its robustness and user-friendliness, enabling us to explore various machine-learning approaches and their combinations simultaneously and automatically. It has been validated in different applications [27, 28]. Furthermore, passing transformer-learned representations to downstream machine-learning approaches for making predictions has been successfully demonstrated [28, 29]. Specifically, we employed AutoGluon’s AutoTabular module, which automates the training and validation process via a stacked ensemble comprising diverse models, including XGBoost, CatBoost, GBM, random forests, and neural networks [30].

We chose the F1 score as a single evaluation metric for model training because it balances precision and recall. The dataset was randomly split into three sets with proportions of 20%, 10%, and 70%, and models were trained on each set using the F1 score along with the best-quality preset. Using the first split dataset (20%), we initially trained a model using all 1280 features. To assess the relative importance of the 1,280 encoder-provided features, we employed the smallest split dataset (10%) for permutation testing, as implemented in the AutoGluon package: In this feature importance test, values for a given feature (column) were randomly shuffled across different residues (rows) and supplied as input to the model. The importance score for each feature is computed by comparing the model's performance on the original dataset with its performance on the permuted datasets. Based on the importance scores derived from the permutation test results of 20 shuffled sets, features were ranked in order of their contribution to the model’s performance. We selected the top *k-ranked* features (*k* = 10, 20, 30, 40, 50, 100, 200, 300, 400, 500, 700, 1000) or all 1280 features for retraining the model on the largest split dataset (70%). The resulting PPI-hot spot prediction model using *k* selected features is referred to as PPI-Hotspot^PLM,*k*^ where the superscript PLM denotes Protein Language Model and the superscript *k* is the number of selected features chosen. To assess whether prediction quality depends on the selection of specific features or the mere number of features, we randomly selected *k* features, instead of selecting them based on permutation testing, and trained the model on the same 70% split dataset used to derive PPI-Hotspot^PLM,*k*^. The resulting PPI-hot spot prediction model using *k* randomly selected features is referred to as PPI-Hotspot^PLM,*k*-random^.

**Fig. 1:**
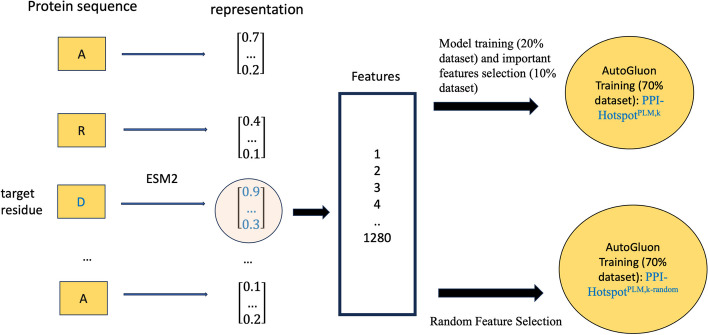
Model training and validation using features derived from the representations learned by the ESM-2 protein language model. The target residue, together with its sequence neighbor aa residues, is passed to the ESM-2 encoder, which produces an *N*-dimensional (*N* = 1280) embedding vector for each residue in the sequence. The 1,280 elements of each vector were supplied as a set of input features for training a model on a 20% split dataset. All features or a reduced set of *k* (*k* < *N*) features were selected either randomly or based on AutoGluon's feature importance test for training a model on a 70% split dataset

To mitigate the influence of outlier values and ensure the stability of our results, the entire procedure depicted in Fig. 1 was repeated a sufficient number of times. For each repetition, a different random seed was used to randomly split the original database into three sets. Across the repetitions, we calculated the average F1 score for each model and compared the differences in averages for the different models to gauge the consistency and stability of the results. We found that 20 repetitions allowed us to detect a statistically significant difference of 0.01 in the F1 score.

### Model training and validation with sequence and structure-based features

**Fig. 2:**
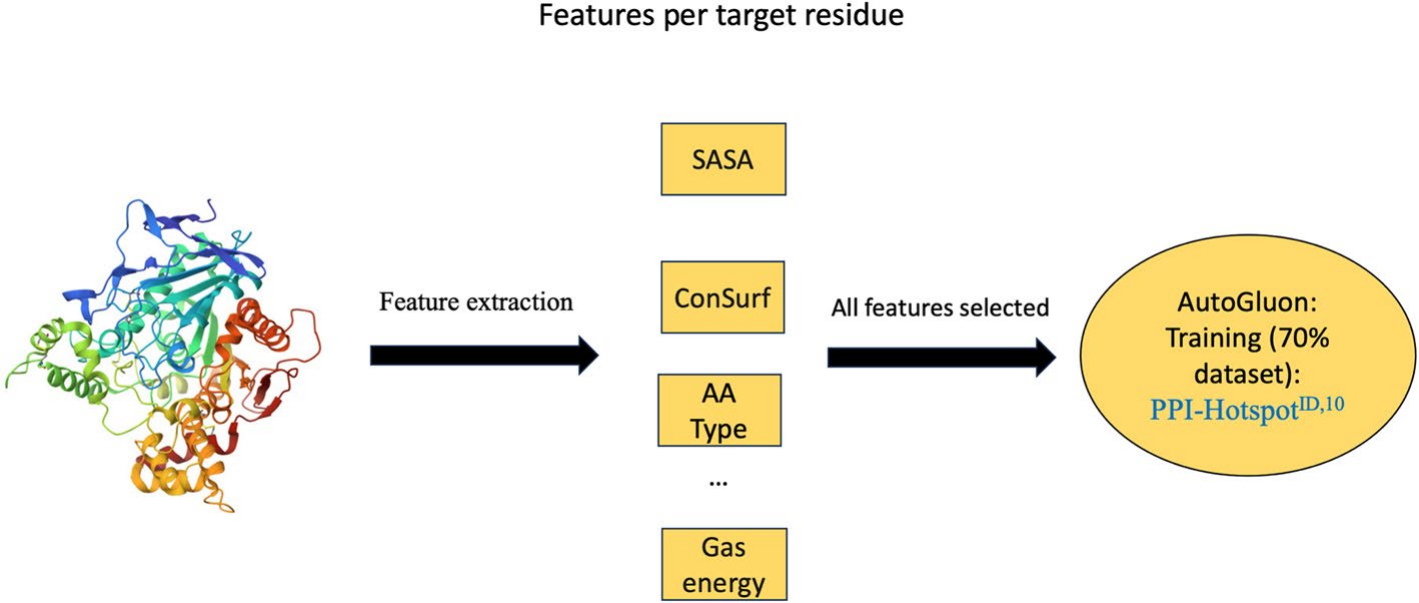
Model training and validation using sequence, structural, and stability attributes. For each residue, we computed the aa type, conservation score, secondary structure, SASA, gas-phase energy and the respective components, as well as the polar and nonpolar solvation free energy. All 10 features were used as input for the AutoGluon training and validation procedure

Instead of using features from protein language models as input, we performed model training and validation on the same 70% split dataset as PPI-Hotspot^PLM,*k*^ using a set of 10 features/residue and the free protein structure as input (Fig. 2). These residue features encompass sequence, structural, and stability attributes including the aa type, conservation score, secondary structure, solvent-accessible surface area (SASA), gas-phase energy and its components, as well as the polar and nonpolar solvation free energy. For each residue, the conservation score was derived from ConSurf [31, 32], which requires search and selection of sequences for multiple sequence alignment. Using the free protein structure, the residue’s secondary structure was based on the DSSP program [33], its SASA was computed using FreeSasa [34], and the per-residue energy/free energy contributions were estimated using the MMPBSA (Molecular Mechanics Poisson-Boltzmann Surface Area) module in AmberTools [35]. For details on the calculations, we direct the reader to the work of Chen et al. [23] The final PPI-hot spot prediction model based on these 10 features is named PPI-Hotspot^ID,10^, where PPI-Hotspot^ID^ is the name of the method for identifying PPI-hot spots using the free protein structure in reference [23] and the superscript 10 indicates that 10 residue features were employed.

## Results

### Performance of PPI-hotspot^PLM,1280^ versus PPI-hotspot^ID,10^

Identifying PPI-hot spots is important for understanding protein function, engineering proteins, and designing PPI modulators. To identify PPI-hot spots, we trained PPI-Hotspot^PLM,*k*^ models using *k* sequence-based features as well as PPI-Hotspot^ID,10^ using 10 residue features on the same 70% split dataset. The mean validation F1 score of the PPI-Hotspot^PLM,1280^ models using all 1280 input features was 0.69 ± 0.018, which is similar to the validation F1 score of 0.71 ± 0.002 achieved by the PPI-Hotspot^ID,10^ (Table 1). However, PPI-Hotspot^ID,10^ required as input the free protein structure to compute the per-residue energy/free energy contributions as well as multiple sequence alignment to compute the conservation score, which may take up to 30 min [23]. In contrast, the encoder generated features in less than a minute. The similarity in validation F1 scores suggests that unsupervised learning can capture the functional properties of residues encoded within aa sequences during evolution.

**Table 1:** Performance of PPI-Hotspot^ID,10^, PPI-Hotspot^PLM,1280^, PPI-Hotspot^PLM,300^, and PPI-Hotspot^PLM,300-random^ on the same 70% split datasets

Method	PPI-Hotspot^ID,10^	PPI-Hotspot^PLM,1280^	PPI-Hotspot^PLM,300^	PPI-Hotspot^PLM,300-random^
Architecture	Ensemble	Ensemble + ESM2	Ensemble + ESM2	Ensemble + ESM2
# of features	10	1280	300	300
Input	Structure	Sequence	Sequence	Sequence
Validation F1-score	0.71 ± 0.002	0.69 ± 0.018	0.71 ± 0.02	0.70 ± 0.016

### Performance of PPI-hotspot^PLM,***k***^ as a function of the number of features,*** k***

**Fig. 3:**
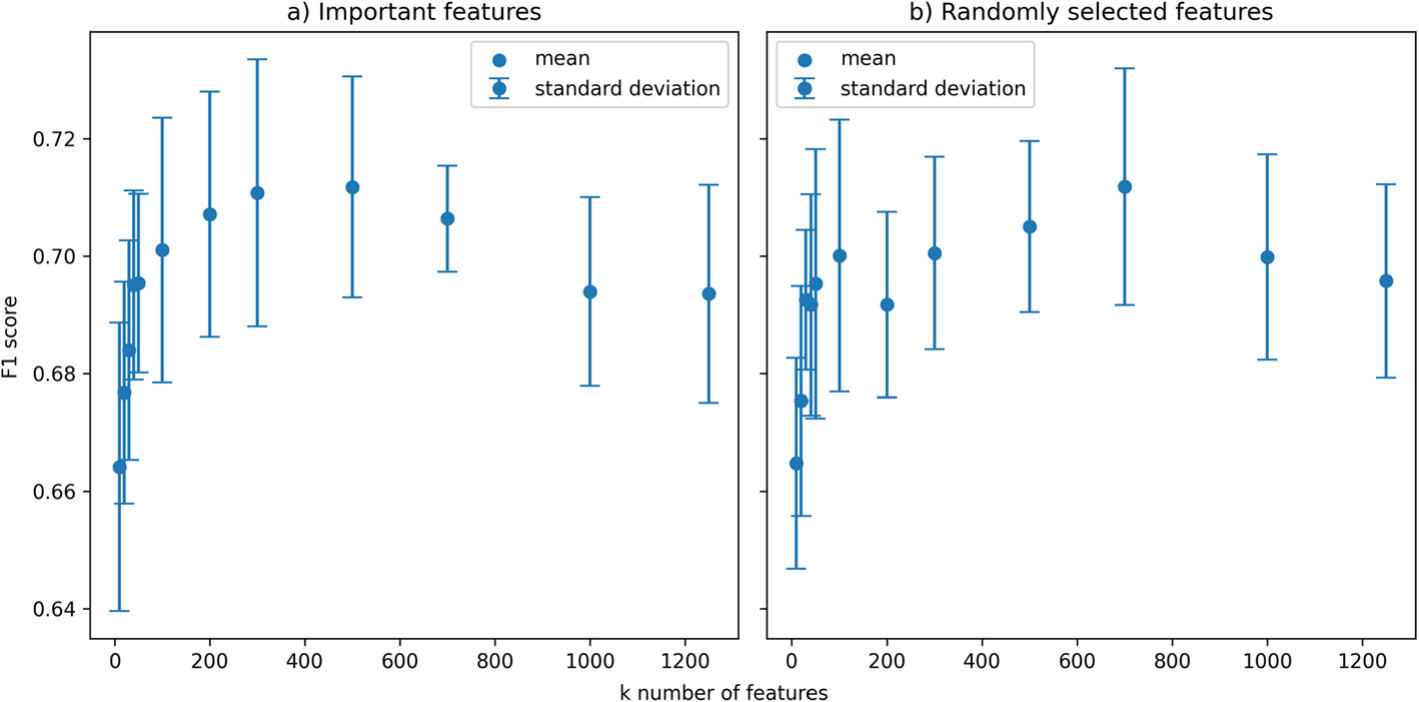
Validation F1 scores for the models trained as a function of the number of features, *k*. Features were selected using either feature importance test (**a**) or randomly (**b**). The dot in the figure corresponds to the mean, whereas the error bar denotes the standard deviation

We examined the effect of selecting the top *k-*ranked features on the performance of the PPI-Hotspot^PLM,*k*^ models with increasing *k*. The results in Fig. 3a show that increasing *k* led to an overall improvement in performance (increase in the F1-score) up to *k* ~ 300. Beyond this threshold, further increases in *k* resulted in a slight decline in the F1 score. We hypothesize that initially, the inclusion of additional important features contributes valuable information, thereby improving the training of the model, but once the number of features greatly exceeds the size of the training dataset, AutoGluon's training routine adopts a more conservative strategy to prevent overfitting, resulting in a slight reduction in final validation precision. We observed a similar trend for randomly selected features, as shown in Fig. 3b.

### Performance of PPI-hotspot^PLM,***k***^ versus PPI-hotspot^PLM,***k***-***random***^

Comparison of the F1 scores from PPI-Hotspot^PLM,*k*^ models using *k* importance-based features and PPI-Hotspot^PLM,*k*-*random*^ models using *k* randomly selected features models (Additional file 1: Table S1) indicates that the number of features *k* plays a crucial role in determining the final model precision. To assess whether the results from importance-based feature selection differ significantly from those of random feature selection we compared the means of F1 scores for a given number of features, *k*, using the statistical t-test. No observable difference was found; e.g., for *k* = 300, the mean F1 scores for PPI-Hotspot^PLM*,300*^ (0.71 ± 0.02) and PPI-Hotspot^PLM,*k*-*random*^ (0.70 ± 0.016) are nearly identical (Table 1). This is consistent with the importance scores obtained from the feature importance test: The features deemed most important contribute no more than 0.03 to the final F1 score. We also attempted to enhance the importance feature test by increasing the number of shuffles in the permutation test. This led to a significant increase in computing time for the importance feature test without any significant improvement in performance compared to random feature selection.

## Discussion

Our primary objective herein was not to develop a highly accurate sequence-based PPI-hot spot prediction method, but rather to showcase the potential of transformer-based protein language models in extracting critical information from sparse datasets whose total number of entries is comparable to or less than the number of elements (N = 1280) in the encoder-generated vector. This is important as experimental collection of certain types of data, such as PPI-hot spots, remains challenging yielding insufficient data for machine learning algorithms to extract meaningful representations. As exemplified herein with elusive PPI-hot spots, protein language models can offer a solution, as their encoder-generated vectors, which encode protein residues and their contexts, provide valuable input features for subsequent machine learning training to predict certain aa residue properties. These input features can be substantially reduced in number through feature importance ranking or random sampling. Our proposed approach is practical for real-world applications: Model training using AutoGluon has proven successful even without GPU support. Although GPU usage is preferred for efficiency, ESM models exhibit sufficient speed to run on CPUs alone, requiring as little as 16 GB of memory. Furthermore, to facilitate ease of use, we provide a notebook in the GitHub repository that runs on Google Colab, enabling users to input their sequences and obtain predicted PPI-hot spots. Note that we sought to provide a fair comparison by using the *same* dataset to compare the PPI-hot spot predictions based on protein language-derived features and those based on features requiring the free protein structure. As other sequence-based PPI-hot spot prediction methods have been trained on different datasets, they could not be fairly compared with our PPI-Hotspot^PLM,1280^ model. Discrepancies in predictions may arise from differences in underlying training datasets, specific features, or machine-learning methodologies.

### Limitations and future work

An evident drawback of the approach outlined here is the lack of a clear explanation as to why specific features are crucial for determining certain residue properties and how they contribute to PPI-hot spot predictions. This limitation aligns with the inherent lack of interpretability of large language models; currently, understanding the inner workings of these models remains elusive. Another limitation is the representativeness of the dataset. Even though our dataset includes data not only from the ASEdb [24] and SKEMPI 2.0 [25] database, but also UniProtKB [26], it is still not comprehensive as experimental studies do not sample all representative protein interactions. Future improvements in model architecture, dataset size (including more experimentally confirmed PPI-hot spots and PPI-nonhot spots), and computational resources may enhance the accuracy of sequence-based PPI-hot spot predictions.

## Conclusions

We have presented a general, robust, and straightforward approach for the training and validation of predictive models using protein language models that can effectively extract valuable information from sparse protein datasets. Specifically, the ESM-2 model showed promising results in predicting PPI-hot spots using all 1,280 features as well as using a subset of features fewer than the number of entries in the dataset. Future improvements in model architecture and increased dataset sizes may further enhance the accuracy of PPI-hot spot predictions, which would aid in understanding protein function and drug design. The ability to do this from just the sequence alone would save time and costs compared to traditional experimental methods. This study further demonstrates that even with sparse datasets, encoder-generated vectors, which encompass residue information and their contexts, offer valuable input features for machine learning to make reliable predictions. In addition, we provide a notebook as part of our source repository, allowing users to run PPI-hot-spot predictions on their protein sequences.

### Supplementary Information

**Additional file 1:** **Supplementary Table S1.** Performance of PPI-Hotspot^PLM,*k*^ and PPI-Hotspot^PLM,*k-random*^ on the same 70% split datasets for all k.

## Data Availability

Supporting code and datasets are available at https://github.com/karsar/PPI_hotspot_seq.
