# Supplementary material for: Using protein language models for protein interaction hot spot prediction with limited data
Source: BMC Bioinformatics. 2024 Mar 16;25:115. doi: 10.1186/s12859-024-05737-2 (PMC10943781; doi:10.1186/s12859-024-05737-2)
Supplement: Supplementary file 1 — Additional file 1: Supplementary Table S1. Performance of PPI-HotspotPLM,k and PPI-HotspotPLM,k-random on the same 70% split datasets for all k. [file 12859_2024_5737_MOESM1_ESM.docx]

**Using Protein Language Models for Protein Interaction Hot Spot Prediction with Limited Data**

Karen Sargsyan^1*^ and Carmay Lim^1*^

^1^Institute of Biomedical Sciences, Academia Sinica, Taipei 115, Taiwan

*Correspondence should be addressed to K. S. (karen.sarkisyan@gmail.com) or C.L. (carmay@gate.sinica.edu.tw)

**Supplementary Table S1**. Performance of PPI-Hotspot^PLM,k^ and PPI-Hotspot^PLM,k-random^ on the same 70% split datasets for all *k*.

| *k* value | PPI-Hotspot^PLM,k^ | PPI-Hotspot^PLM,k-random^ |
| --- | --- | --- |
| 10 | 0.664 ± 0.024 | 0.664 ± 0.018 |
| 20 | 0.677 ± 0.018 | 0.675 ± 0.019 |
| 30 | 0.684 ± 0.019 | 0.692 ± 0.011 |
| 40 | 0.695 ± 0.016 | 0.691 ± 0.019 |
| 50 | 0.695 ± 0.015 | 0.695 ± 0.023 |
| 100 | 0.701 ± 0.023 | 0.700 ± 0.023 |
| 200 | 0.707 ± 0.021 | 0.691 ± 0.016 |
| 300 | 0.710 ± 0.023 | 0.700 ± 0.016 |
| 500 | 0.711 ± 0.019 | 0.705 ± 0.014 |
| 700 | 0.706 ± 0.010 | 0.711 ± 0.02 |
| 1000 | 0.694 ± 0.016 | 0.699 ± 0.017 |
| 1280 | 0.693 ± 0.018 | 0.693 ± 0.018 |
